# Advanced Dynamic Cell and Tissue Culture

**DOI:** 10.3390/bioengineering5030065

**Published:** 2018-08-11

**Authors:** Jan Hansmann, Dominik Egger, Cornelia Kasper

**Affiliations:** 1 Department of Tissue Engineering and Regenerative Medicine, University Hospital Wuerzburg, Roentgenring 11, 97070 Wuerzburg, Germany; 2 Translational Center Regenerative Therapies, Fraunhofer Institute for Silicate Research ISC, Roentgenring 11, 97070 Wuerzburg, Germany; 3 Department of Biotechnology, University of Natural Resources and Life Sciences, Muthgasse 18, 1190 Vienna, Austria; dominik.egger@boku.ac.at

In order to meet “modern” cell culture requirements, strategies have to be developed which mimic the in vivo cellular environment. Although it is known that static 2D cell culture systems resemble the complexity of the physiologic conditions only to a limited extend, the expansion and differentiation of cells on plastic surfaces is still “gold standard” in many cell culture applications. In contrast to static culture conditions, the use of bioreactors bears the potential to achieve a more organ- or tissue-specific dynamic culture by providing physical cues and improved nutrient supply [1]. However, scalability, integration of biosensors, a suitable level of automation, as well as the identification of optimal culture vessels, need to be considered when developing a bioreactor system. In addition to tissue-specific physical cues and a suitable nutrient supply, also the interaction with the extracellular matrix has a strong impact on the development of the cultured cells, and thus introduces novel design criteria for optimal culture substrates even in the third dimension. Moreover, the connection of several cell types or tissues in a microfluidic system allows cell-cell crosstalk and thereby complements the cell-substrate interaction. In three reviews and three research articles, this special issue provides a detailed overview on current trends and developments in advanced cell and tissue culture technologies.

The review by Egger et al. [2] comprehends recent insights into the culture of mesenchymal stem cells. Due to their high regenerative potential, mesenchymal stem cells are considered as promising candidates for cell-based therapies. To improve the therapeutic effect, for example, a robust stemness or an increased survival rate after implantation, control of the culture conditions such as oxygen tension, three-dimensional arrangement, or mechanical shear stress can be harnessed. By focusing on the culture of mesenchymal stem cells as three-dimensional aggregates in suspension bioreactor systems, this review discusses novel concepts for an efficient scalable bulk expansion of mesenchymal stem cells or mesenchymal-stem-cell-derived extracellular vesicles.

In addition to an improved and scalable generation of a sufficient number of functional cells for cell therapies the three-dimensional culture of stem cells also bears potential for a predictive and cost-efficient assessment of novel drug candidates. The review by Miranda et al. [3] presents current progress in the generation of three-dimensional organoids from human pluripotent stem cells. Moreover, limitations are discussed and technologies for the characterization of the organoids after the administration of a drug are presented. With the possibility to test drugs with patient-derived organoids, the article also addresses aspects of personalized medicine.

A practical example for a test system to assess drug candidates is described in the article written by Schimek et al. [4]. Here, a full-thickness skin equivalent in a 96-well insert format is presented. The skin model supports the culture in a microfluidic two-organ chip that comprises a micro pump as well as a fluidic connection to transport cell culture medium and to facilitate permeation assays. Moreover, tissue-specific culture conditions at the air-liquid-interface are possible. By the use of fluorescein sodium salt, a time-dependent permeation capacity was detected for fluorescein and the feasibility for permeation assays in the setup is demonstrated. The experiments are further investigated by computational fluid dynamic modeling, allowing a deeper understanding of the permeation process and the prediction of long-term results. Due to the minimized format, the culture in a scalable microfluidic device, and the in silico approach, the technology paves the way for mid- to high-throughput testing.

A microfluidic system is also used in the study described by Pérez-Rodríguez et al. [5]. By chemotaxis, fibroblast are recruited to a wound in order to support tissue regeneration. However, the migration of fibroblasts has hardly been studied, especially in the light of a specific fibroblast origin. In this article, the migration patterns of dermal and cardiac fibroblasts are compared after application of different stimuli. Thereby, the authors found different responses between the two fibroblast cultures indicating an origin-related intracellular regulation.

In both studies described above, hydrogels were applied as three-dimensional matrix. A suitable tool to efficiently synthesize hydrogels with a specific three-dimensional shape is presented by the article of Pepelanova et al. [6]. Based on gelatin-methacryloyl, a physiological microenvironment for human adipose-tissue-derived mesenchymal stem cells is created. To achieve a mechanically stable tissue construct, different compositions of the hydrogel and different polymerization conditions are compared. In addition, the supplementation of biocompatible additives facilitates the modification of the hydrogel viscosity. This may result in an improve applicability of the system for bioprinting.

A comprehensive summary, which addresses most topics of the articles in the special issue, is provided by the review of Yuan et al. [7]. The review presents technologies to fabricate three-dimensional scaffolds and discusses current limitations. Hereby, a strong focus is given on bioprinting. Moreover, vibration is considered as an elegant way to introduce mechanical stimulation in a cell culture. The authors also picture future trends in scaffold generation and dynamic cell culture systems, envisioning three-dimensional-printed scaffolds with local mechanical stimulation.

With the first volume of this special issue, the editors want to emphasize the important role of modern cell culture technologies in biotechnological applications and regenerative medicine. While the included reviews allow a technological assessment of the advances in the field, the research articles give practical examples. Finally, the editors thank the authors for their appreciated contributions. 
